# Supplementary material for: Survey of Flemish Poultry Farmers on How Birds Fit for Transport to the Slaughterhouse Are Selected, Caught, and Crated and Their Opinions Regarding the Pre-Transport Process
Source: Animals (Basel). 2024 Nov 12;14(22):3241. doi: 10.3390/ani14223241 (PMC11591035; doi:10.3390/ani14223241)
Supplement: Supplementary file 1 [file animals-14-03241-s001.zip › animals-3268699-supplementary.pdf]

SURVEY POULTRY FARMERS

Introduction

1. Are you:

   a. A professional broiler farmer (go to point 2)
   b. A professional laying hen farmer (go to point 3)

2. In which province(s) is your farm located?
   a. West Flanders
   b. East Flanders
   c. Brussels
   d. Antwerp
   e. Flemish Brabant
   f. Limburg

3. What is your age?
   a. < 18 years
   b. 18 - 25 years
   c. 25 - 35 years
   d. 35 - 45 years
   e. 45 - 55 years
   f. 55 - 65 years
   g. > 65 years

4. What is your gender?
   a. Male
   b. Female
   c. X

5. What is your level of education?
   a. Secondary diploma
   b. College diploma
   c. University diploma
   d. Other (please specify)

Flemish Broiler Farmers (based on question 1, you know if they keep broilers)

Focus on the last time the broilers were caught at the end of the round on your farm.

1. How many barns were caught last time on your farm + number of animal spaces per barn?

2. What genetic line/hybrid were your broilers?

3. What was the slaughter age of the broilers?
   a. Partial pickers: 0 to … days (slider)
   b. Full pickers: 0 to … days (slider)

4. What was the weight of the broilers?
   a. Partial pickers: 0 to … kg (slider)
   b. Full pickers: 0 to … kg (slider)

Selection

Focus on the last time the broilers were caught at the end of the round on your farm.

Normal selection

1. How often was the selection of animals applied during the round?
   a. X times per day (fill in)
   b. X times per week (fill in)
2. Who selected the animals?
   a. Farm veterinarian
   b. Poultry farmer
   c. Staff
   d. Other (please specify)
3. How long did the selection of animals take?
   a. 0 to ... hours (slider)
4. Were sick or weak chickens, besides dead chickens, also removed/selected?
   a. Yes
   b. No
5. For what reasons were the animals selected? (if yes)
   a. Condition + number of animals
6. What are the challenges/disadvantages of selecting animals?
   a. Cost
   b. Time
   c. Lack of knowledge about which animals to select
   d. Other ...
7. What are the advantages of selecting animals?
   a. Animal welfare
   b. Less rejection at the slaughterhouse
   c. More uniform animals
   d. Sick animals do not infect others
   e. Less feed waste
   f. Other ...
8. Do you have suggestions to optimize animal selection? If yes, what are they?
   a. Yes
   b. No
9. Did you do an extra selection just before loading?

Extra selection just before loading (if yes)

1. How many hours before the start of the catching and loading process was the final selection of animals unsuitable for transport?
   a. 0 to ... hours (slider)
2. How long did the selection of animals unsuitable for transport take?
   a. 0 to ... hours (slider)

3. Did you proceed differently during the last selection round?
   a. Yes + clarification (fill in)
   b. No

Catching and Loading

Focus on the last time the broilers were caught at the end of the round on your farm.

1. What were the start and end times and date of the last catching and loading (from the moment the catching team entered the barn to the departure of the last truck)?
   a. Start time (fill in)
   b. End time (fill in)
   c. Date (fill in)
2. What preparations were made before the catching and loading process?
   a. Feed removal + time
   b. Water removal + time
   c. How were the lights programmed during catching and loading, did you change them? + time
   d. How were the climate and ventilation programmed during catching and loading, did you change them? + time
   e. Other (fill in) + time
3. What do you think is the best time to catch and load chickens? Why?
4. How were the animals caught last time?
   a. Manually
   b. Mechanically

Manual Catching (if manually selected)

Focus on the last time the broilers were caught at the end of the round on your farm.

1. How many chickens were taken in both hands?
2. Were the chickens caught by 1 or 2 legs?
   a. 1
   b. 2
3. Was the method of manual catching your own choice, or was it imposed?
   a. Own choice. Why do you prefer this method?
   b. Imposed. By whom?
4. If the manual catching method was not your own choice, what other method would you prefer and why? (only for those who indicated "imposed" in the previous question)
5. Did you use an external catching team?
   a. Yes, why?
   b. No, why?
6. Which catching team was used during catching and loading? (if external catching team selected)
7. Is this catching team GSP (Good Service Practices) recognized? (if external catching team selected)

a. Yes
b. No

8. Were you (the poultry farmer) present during the catching and loading process?
a. Yes, from start to finish
b. Only at the beginning
c. Only in the middle
d. Only at the end
e. No
f. Other (fill in)

9. What did you do during the catching and loading process? (if yes, only at the beginning or partially indicated)
a. Help catch
b. Supervise
c. Instruct
d. Other (fill in)

10. How satisfied were you with the catching and loading process?
a. 0 to 100% (slider)

11. If you would like to elaborate on your satisfaction, you can do so here.

12. What aspects were considered during catching and loading?

13. To what extent do you think the welfare of the chickens was guaranteed during the catching and loading process?
a. 0 to 100% (slider)

14. To what extent do you find the following aspects of catching and loading burdensome for the catchers?
a. Physically (0 to 100% slider)
b. Mentally (0 to 100% slider)

15. To what extent do you think the type of container influences animal mortality and/or welfare in general?
a. Not at all/slightly/neutral/quite/absolutely (scale)

16. Do you have any suggestions to improve manual catching? If yes, what are they (animal welfare/ergonomics, etc.)?

17. Rank the following catching methods according to the one you would most prefer to apply:
a. 2 chickens in hand + by 1 leg
b. 2 chickens in hand + by 2 legs
c. 3 chickens in hand + by 1 leg
d. 3 chickens in hand + by 2 legs
e. > 3 chickens in hand + by 1 leg
f. > 3 chickens in hand + by 2 legs
g. 1 chicken caught upright
h. 2 chickens caught upright
i. By the wings

18. Rank the following catching methods according to the best method for the welfare of the animal:
    a. 2 chickens in hand + by 1 leg
    b. 2 chickens in hand + by 2 legs
    c. 3 chickens in hand + by 1 leg
    d. 3 chickens in hand + by 2 legs
    e. > 3 chickens in hand + by 1 leg
    f. > 3 chickens in hand + by 2 legs
    g. 1 chicken caught upright
    h. 2 chickens caught upright
    i. By the wings

19. Rank the following catching methods according to the best method for the welfare of the catcher:
    a. 2 chickens in hand + by 1 leg
    b. 2 chickens in hand + by 2 legs
    c. 3 chickens in hand + by 1 leg
    d. 3 chickens in hand + by 2 legs
    e. > 3 chickens in hand + by 1 leg
    f. > 3 chickens in hand + by 2 legs
    g. 1 chicken caught upright
    h. 2 chickens caught upright
    i. By the wings

The Catching Team

Focus on the last time broilers were caught at the end of the round on your farm.

1. How efficient was communication with the catching team?
   a. 0 to 100% (slider)

2. How many people were on the catching team?
   a. 0 to 40 (slider)

3. To what extent did the following problems occur during manual catching?
   a. Excessive noise (0 to 100% slider)
   b. Animal stress and agitation (0 to 100% slider)
   c. Animal injuries from catching (0 to 100% slider)
   d. Inappropriate handling of animals (0 to 100% slider)
   e. Inefficiency & slowness (0 to 100% slider)
   f. Uneven distribution of chickens in containers (0 to 100% slider)

4. How is the catching team paid? You may freely indicate the exact amount.
   a. Per chicken (exact amount)
   b. Per barn (exact amount)
   c. Per hour (exact amount)
   d. Other (please specify) (exact amount)

5. Would you prefer a different payment system and why?

Mechanical Catching (if selected)

Focus on the last time broilers were caught at the end of the round on your farm.

1. What type of catching machine (manufacturer) was used?
   a. Chicken Cat Harvester
   b. Super Apollo
   c. ...
2. How many people were on the catching team, besides those operating the machine?
   a. 0 to 40 (slider)
3. How efficient was communication with the catching team?
   a. 0 to 100% (slider)
4. Were you (the poultry farmer) present during the entire catching and loading process?
   a. Yes, from start to finish
   b. Only at the beginning
   c. Only in the middle
   d. Only at the end
   e. No
   f. Other (please specify)
5. What did you do during the catching and loading process?
   (if yes, only at the beginning or partially indicated)
   a. Help catch
   b. Supervise
   c. Instruct
   d. Other (please specify)
6. Were there any technical or other (unusual) malfunctions? If yes, what were they?
7. What percentage of the animals was cleared with the catching machine?
   a. 0 to 100% (slider)
8. To what extent did the following problems occur during mechanical catching?
   a. Excessive noise (0 to 100% slider)
   b. Animal stress and agitation (0 to 100% slider)
   c. Animal injuries from catching (0 to 100% slider)
   d. Inappropriate handling of animals (0 to 100% slider)
   e. Inefficiency & slowness (0 to 100% slider)
   f. Uneven distribution of chickens in containers (0 to 100% slider)
9. What are the advantages of mechanical catching?
   a. Fewer workers needed
   b. Less stress for the animals
   c. Other (please specify)
10. How satisfied were you with the catching and loading process?
    a. 0 to 100% (slider)
11. If you would like to elaborate on your satisfaction, you can do so here.
12. What was the total cost of mechanical catching?
    a. Price of the catching team

b. Price of the catching machine
c. Other (please specify) + price

13. Do you have suggestions for improving mechanical catching? If yes, what are they (animal welfare/ergonomics, etc.)?

14. To what extent do you think the type of container influences animal mortality and/or welfare in general?
a. Not at all/slightly/neutral/quite/absolutely (scale)

15. Rank the following catching methods according to the one you would most prefer to apply:
a. 2 chickens in hand + by 1 leg
b. 2 chickens in hand + by 2 legs
c. 3 chickens in hand + by 1 leg
d. 3 chickens in hand + by 2 legs
e. > 3 chickens in hand + by 1 leg
f. > 3 chickens in hand + by 2 legs
g. 1 chicken caught upright
h. 2 chickens caught upright
i. By the wings
j. Mechanical catching

16. Rank the following catching methods according to the best method for the welfare of the animal:
a. 2 chickens in hand + by 1 leg
b. 2 chickens in hand + by 2 legs
c. 3 chickens in hand + by 1 leg
d. 3 chickens in hand + by 2 legs
e. > 3 chickens in hand + by 1 leg
f. > 3 chickens in hand + by 2 legs
g. 1 chicken caught upright
h. 2 chickens caught upright
i. By the wings
j. Mechanical catching

17. Rank the following catching methods according to the best method for the welfare of the catcher:
a. 2 chickens in hand + by 1 leg
b. 2 chickens in hand + by 2 legs
c. 3 chickens in hand + by 1 leg
d. 3 chickens in hand + by 2 legs
e. > 3 chickens in hand + by 1 leg
f. > 3 chickens in hand + by 2 legs
g. 1 chicken caught upright
h. 2 chickens caught upright
i. By the wings
j. Mechanical catching

Flemish Laying Hen Farmers

(Based on question 1, you know if they keep laying hens)

Focus on the last time the laying hens were caught at the end of the round on your farm.

1. In which system(s) were the laying hens that were last caught on your farm housed?
   i. Without free range

   1. Floor housing system
      a. Number of barns?
      b. Number of animal spaces per barn?
      c. With or without winter garden?

   2. Aviary system
      a. Number of barns?
      b. Number of animal spaces per barn?
      c. With or without winter garden?

ii. With free range

   3. Organic or not?

   4. Floor housing system
      a. Number of barns?
      b. Number of animal spaces per barn?
      c. With or without winter garden?

   5. Aviary system
      a. Number of barns?
      b. Number of animal spaces per barn?
      c. With or without winter garden?

   6. Mobile housing
      a. Number of barns?
      b. Number of animal spaces per barn?
      c. With or without winter garden?

2. What breed(s) of laying hen(s) were last caught on your farm?

3. What was the slaughter age of the laying hens (in weeks)?

Selection

Focus on the last time the laying hens were caught at the end of the round on your farm.

Normal selection

1. How often was the selection of animals applied during the round?
   a. X times per day (fill in)
   b. X times per week (fill in)

2. Who selected the animals?
   a. Farm veterinarian
   b. Poultry farmer
   c. Staff
   d. Other (please specify)

3. How long did the selection of animals take?
   a. 0 to … hours (slider)

4. Were sick or weak chickens, besides dead chickens, also removed/selected?
   a. Yes
   b. No

5. For what reasons were the animals selected? *(if yes)*
   a. Condition + number of animals

6. What are the challenges/disadvantages of selecting animals?
   a. Cost
   b. Time
   c. Lack of knowledge about which animals to select
   d. Other …

7. What are the advantages of selecting animals?
   a. Animal welfare
   b. Less rejection at the slaughterhouse
   c. More uniform animals
   d. Sick animals do not infect others
   e. Less feed waste
   f. Other …

8. Do you have suggestions to optimize animal selection? If yes, what are they?
   a. Yes
   b. No

9. Did you do an extra selection just before loading?

Extra selection just before loading (if yes)

1. How many hours before the start of the catching and loading process was the final selection of animals unsuitable for transport?
   a. 0 to … hours (slider)

2. How long did the selection of animals unsuitable for transport take?
   a. 0 to … hours (slider)

3. Did you proceed differently during the last selection round?
   a. Yes + clarification (fill in)
   b. No

Catching and Loading

Focus on the last time the laying hens were caught at the end of the round on your farm.

1. What were the start and end times and date of the last catching and loading (from the moment the catching team entered the barn to the departure of the last truck)?
   a. Start time (fill in)
   b. End time (fill in)
   c. Date (fill in)

2. What preparations were made before the catching and loading process?
   a. Feed removal + time
   b. Water removal + time
   c. How were the lights programmed during catching and loading, did you change them? + time
   d. How were the climate and ventilation programmed during catching and loading, did you change them? + time
   e. Manure removal + time
   f. Closing gates under aviary system + time
   g. Closing laying nests + time
   h. Other (fill in) + time

3. What do you think is the best time to catch and load chickens? Why?

Manual Catching

Focus on the last time the laying hens were caught at the end of the round on your farm.

1. How many chickens were taken in both hands?

2. Were the chickens caught by 1 or 2 legs?
   a. 1
   b. 2

3. Was the method of manual catching your own choice, or was it imposed?
   a. Own choice, why do you prefer this method?
   b. Imposed, by whom?

4. If the manual catching method was not your own choice, what other catching method would you prefer and why? *(only for those who indicated "imposed" in the previous question)*

5. Did you use an external catching team?
   a. Yes, why?
   b. No, why?

6. Which catching team was used during catching and loading? *(If external catching team indicated)*

7. Is this catching team GSP (Good Service Practices) recognized? *(If external catching team indicated)*
   a. Yes
   b. No

8. Were you (the poultry farmer) present during the catching and loading process?
   a. Yes, from start to finish
   b. Only at the beginning
   c. Only in the middle
   d. Only at the end
   e. No
   f. Other (please specify)

9. What did you do during the catching and loading process? *(if yes, only at the beginning or partially indicated)*
   a. Help catch
   b. Supervise
   c. Instruct
   d. Other (fill in)
10. How satisfied were you with the catching and loading process?
    a. 0 to 100% (slider)
11. If you would like to elaborate on your satisfaction, you can do so here.
12. What aspects were considered during catching and loading?
13. To what extent do you think the welfare of the chickens was guaranteed during the catching and loading process?
    a. 0 to 100% (slider)
14. To what extent do you find the following aspects of catching and loading burdensome for the catchers?
    a. Physically (0 to 100% slider)
    b. Mentally (0 to 100% slider)
15. To what extent do you think the type of container influences animal mortality and/or welfare in general?
    a. Not at all/slightly/neutral/quite/absolutely (scale)
16. Do you have suggestions to improve manual catching? If yes, what are they (animal welfare/ergonomics, etc.)?
17. Rank the following catching methods according to the one you would most prefer to apply:
    a. 2 chickens in hand + by 1 leg
    b. 2 chickens in hand + by 2 legs
    c. 3 chickens in hand + by 1 leg
    d. 3 chickens in hand + by 2 legs
    e. > 3 chickens in hand + by 1 leg
    f. > 3 chickens in hand + by 2 legs
    g. 1 chicken caught upright
    h. 2 chickens caught upright
    i. By the wings
18. Rank the following catching methods according to the best method for animal welfare:
    a. 2 chickens in hand + by 1 leg
    b. 2 chickens in hand + by 2 legs
    c. 3 chickens in hand + by 1 leg
    d. 3 chickens in hand + by 2 legs
    e. > 3 chickens in hand + by 1 leg
    f. > 3 chickens in hand + by 2 legs
    g. 1 chicken caught upright
    h. 2 chickens caught upright
    i. By the wings

19. Rank the following catching methods according to the best method for the welfare of the catcher:
    a. 2 chickens in hand + by 1 leg
    b. 2 chickens in hand + by 2 legs
    c. 3 chickens in hand + by 1 leg
    d. 3 chickens in hand + by 2 legs
    e. > 3 chickens in hand + by 1 leg
    f. > 3 chickens in hand + by 2 legs
    g. 1 chicken caught upright
    h. 2 chickens caught upright
    i. By the wings

The Catching Team

Focus on the last time the laying hens were caught at the end of the round on your farm.

1. How efficient was communication with the catching team?
   a. 0 to 100% (slider)

2. How many people were on the catching team?
   a. 0 to 40 (slider)

3. To what extent did the following problems occur during manual catching?
   a. Excessive noise (0 to 100% slider)
   b. Animal stress and agitation (0 to 100% slider)
   c. Animal injuries from catching (0 to 100% slider)
   d. Inappropriate handling of animals (0 to 100% slider)
   e. Inefficiency & slowness (0 to 100% slider)
   f. Uneven distribution of chickens in containers/crates (0 to 100% slider)

4. How is the catching team paid? You may freely indicate the exact amount.
   a. Per chicken (exact amount)
   b. Per barn (exact amount)
   c. Per hour (exact amount)
   d. Other (please specify) (exact amount)

5. Would you prefer a different payment system and why?
